# People With Low Back Pain Display a Different Distribution of Erector Spinae Activity During a Singular Mono-Planar Lifting Task

**DOI:** 10.3389/fspor.2019.00065

**Published:** 2019-12-20

**Authors:** Andy Sanderson, Corrado Cescon, Nicola R. Heneghan, Pauline Kuithan, Eduardo Martinez-Valdes, Alison Rushton, Marco Barbero, Deborah Falla

**Affiliations:** ^1^Centre of Precision Rehabilitation for Spinal Pain (CPR Spine), School of Sport, Exercise and Rehabilitation Sciences, University of Birmingham, Birmingham, United Kingdom; ^2^Rehabilitation Research Laboratory 2rLab, Department of Business Economics Health and Social Care, University of Applied Sciences and Arts of Southern Switzerland, Manno, Switzerland

**Keywords:** high density EMG, muscle activity, EMG, low back pain, ergonomics

## Abstract

This study aimed to investigate the variation in muscle activity and movement in the lumbar and lumbothoracic region during a singular mono-planar lifting task, and how this is altered in individuals experiencing low back pain (LBP). Muscle activity from the lumbar and lumbothoracic erector spinae of 14 control and 11 LBP participants was recorded using four 13 × 5 high-density surface electromyography (HDEMG) grids. Root mean squared HDEMG signals were used to create spatial maps of the distribution of muscle activity. Three-dimensional kinematic data were recorded focusing on the relationship between lumbar and thoracic movements. In the task, participants lifted a 5 kg box from knee height to sternal height, and then returned the box to the starting position. The center of muscle activity for LBP participants was found to be systematically more cranial throughout the task compared to the control participants (*P* < 0.05). Participants with LBP also had lower signal entropy (*P* < 0.05) and lower absolute root mean squared values (*P* < 0.05). However, there were no differences between groups in kinematic variables, with no difference in contributions between lumbar and thoracic motion segments (*P* > 0.05). These results indicate that participants with LBP utilize an altered motor control strategy to complete a singular lifting task which is not reflected in their movement strategy. While no differences were identified between groups in the motion between lumbar and thoracic motion segments, participants with LBP utilized a less homogenous, less diffuse and more cranially focussed contraction of their erector spinae to complete the lifting movement. These results may have relevance for the persistence of LBP symptoms and the development of new treatments focussing on muscle retraining in LBP.

## Introduction

Globally, low back pain (LBP) is a leading cause of disability and has been since this metric was first reported in 1990 (James et al., 2018). Recent studies have suggested that there is a worldwide point prevalence of activity-limiting LBP of 560 million people (Hartvigsen et al., 2018). There is a large economic cost associated with the treatment of LBP and in the UK alone, the direct annual healthcare costs of chronic LBP are estimated as £1.8–2.3 billion (Hong et al., 2013). In the USA, the wider economic impact of LBP is thought to be between $100–200 billion per annum (Duthey, 2013).

The prevalence and persistence of LBP is believed to be in part driven by exposure to manual handling, for instance lifting heavy weights in occupational tasks, or assisting moving patients in healthcare professions (Wai et al., 2010; Andersen et al., 2017; Silvetti et al., 2019). A meta-review of eight previous systematic reviews considering the effect of occupational tasks on LBP was conducted in 2011; identifying manual handling as a causal factor for LBP symptoms, whereas conflicting evidence existed for lifting (Kwon et al., 2011). Nonetheless, in more contemporary studies, occupational or routine lifting have been identified as a risk factor for the persistence of LBP symptoms (Andersen et al., 2017; Brandt et al., 2018). Most studies which investigate lifting in people with LBP have focused on repeated lifting movements, and while this is reflective of some occupational tasks, there are occupations which involve less frequent lifting which may still be relevant for the initiation and/or perpetuation of LBP. For example, several studies have investigated links between manual handling and LBP in nurses (Stobbe et al., 1988; Byrns et al., 2004), and while it is acknowledged that this is not a primary role of a nurse, the evidence supports a link between this occasional lifting and LBP.

Surface electromyography (EMG) can be used to evaluate the effects of both chronic and acute pain on muscle behavior (Madeleine et al., 2006; Lariviere et al., 2011; Falla et al., 2017) and classic bipolar EMG has been used extensively to quantify the effect of chronic or persistent LBP on muscle activity during lifting (Fabian et al., 2005; Ershad et al., 2009; Correia et al., 2016). In more recent studies, spurred on by advances in EMG technology, high-density EMG (HDEMG) has been used to evaluate changes in the spatial distribution of back muscle activity in people with LBP (Abboud et al., 2014; Falla et al., 2014; Martinez-Valdes et al., 2019; Sanderson et al., 2019). These studies have identified differences in the topographical distribution of erector spinae (ES) activity in people with LBP, including reduced re-distribution of activity during both dynamic and static tasks (Abboud et al., 2014; Falla et al., 2014).

HDEMG has also been applied to specifically evaluate lumbar muscle activity in people with and without LBP during lifting (Falla et al., 2014, 2017). In a study which investigated the spatial distribution of ES activity during a repetitive mono-planar lifting task (Falla et al., 2014), participants with LBP were seen to have reduced variation in the location of the centroid of the EMG amplitude map, indicating less re-distribution of muscle during this task. Although the study revealed an altered distribution of lumbar ES activity in those with LBP, the findings were limited to the specific region evaluated (i.e., lumbar ES unilaterally spanning a region from ~L2 to L5). The findings were also potentially influenced by fatigue since the task was performed repetitively. In this study, participants with LBP were found to be less able to counter localized fatigue and redistribute activity to regions of the muscle which were not previously as active, instead repeatedly activating more cranial portions of the investigated region. It is possible that the cranial distribution and shift in activity identified in the LBP group could be due to the engagement of the thoracolumbar regions of the ES, which were not investigated. Similarly, this cranial focus of activity in participants with LBP was also identified in a static endurance task, with LBP participants focussing activation in only the more cranial regions of the assessed area (Sanderson et al., 2019). Several previous studies have investigated dissociation between lumbar and thoracolumbar contributions to movements in LBP populations (Langevin et al., 2011; Elgueta-Cancino et al., 2014), however the muscular contributions of these regions have not previously been measured.

Here we extend previous work by performing a comprehensive mapping of both lumbar and thoracolumbar muscle activity during a single repetition of a mono-planar lifting task in order to specifically examine the influence of LBP in the absence of a fatiguing task. Additionally, we record spinal kinematics in a segmental arrangement (Muller et al., 2016) to quantify differences between movement in the lumbar and the thoracolumbar regions of the spine.

Specifically, the aim of this study was to apply HDEMG bilaterally over the lumbar and thoracolumbar ES and utilize segmental spinal 3D kinematics to assess for potential differences in the spatial distribution of ES muscle activity and movement during a singular lifting task. We hypothesized that LBP participants would display a different distribution of ES activity during the lifting task characterized by a shift of activity away from the lumbar region toward the thoracolumbar region. Additionally, we hypothesized that this difference in muscle behavior would be reflected in a different movement strategy characterized by less lumbar spinal motion in those with LBP.

## Methods

This study was an observational cross-sectional study, conducted at the Centre of Precision Rehabilitation for Spinal Pain at the University of Birmingham between 2017 and 2018.

### Participants

Participants aged 18–65 were recruited via poster and social media advertisements from the students, staff, and community of the University of Birmingham. Participants with LBP were considered for this study if they had experienced LBP symptoms for more than half of the days of the previous 6 months (Dionne et al., 2008), and were not under current healthcare management for their LBP (a requirement of the ethical committee). Participants for whom LBP symptoms were related to trauma, fractures, or spinal stenosis were excluded. Additionally, the presence of radiating pain down the leg was an exclusion criterion. Age- and gender-matched control participants were recruited who had no history of low back or lower limb pain. Participants from both groups were excluded if they were on high doses of anti-inflammatories (>30 mg morphine equivalent dose), were pregnant, or were experiencing any concurrent systemic, rheumatic or neuro-musculoskeletal disorders which could confound testing.

### Questionnaires

Prior to commencing data collection, participants were asked to complete several questionnaires in order to assess their baseline characteristics. A bespoke back pain questionnaire was used to collect information relating to the characteristics, duration, and severity of participants LBP. Within this questionnaire, participants were asked to rate their current and recent levels of LBP on an 11-point numerical scale (Breivik et al., 2008). The general health of the sample was assessed using the SF-36 (V2), which has been shown to be a reliable measure of physical and mental health (Walsh et al., 2003). The International Physical Activity Questionnaire (IPAQ) was used to gain metrics on the level of physical activity within the participant cohorts in the week prior to data collection (Craig et al., 2003). The level of disability within the groups was assessed using the Oswestry Disability Index (ODI), a questionnaire which has previously been shown to be reliable in samples with lower levels of disability, as was anticipated (Fairbank and Pynsent, 2000). In order to assess any fear surrounding movement or relating to their level of pain, the participants were asked to complete the Fear Avoidance Beliefs Questionnaire (FABQ) and the Pain Catastrophizing Scale (Waddell et al., 1993; Osman et al., 1997). The Depression, Anxiety and Stress Scale (DASS-21) was also completed to identify the levels of mental health within the groups (Lovibond and Lovibond, 1995; Henry and Crawford, 2005).

### Experimental Task

**Figure 1:**
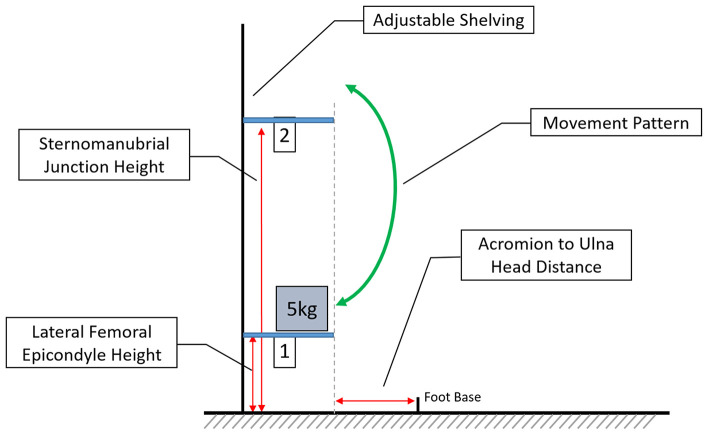
Depicting the experimental set-up, with adjustable height shelving and the anthropomorphic characteristics which determine height and placement. Foot base describes the midpoint of the feet and the arrow indicates the movements between shelf 1 and shelf 2 (not to scale).

The experimental task consisted of lifting a 5 kg weighted box between two shelves situated anterior to the participant, simulating an occupational lifting task (Falla et al., 2014). To ensure the correct adjustment of shelf height and distance from the subject, several anatomical features were identified by palpation and measured on each participant. The lower shelf (S1) was set at the height of the lateral femoral epicondyle, the upper (S2) at the height of the sternomanubrial junction. Measured from the midpoint of the feet, the shelving was placed the distance from the acromion to the ulna head anterior to the participant (Figure 1).

To complete the task, subjects were required to stand in a quiet standing position, with their heels 17 cm apart and feet at a 14° angle to each other (McIlroy and Maki, 1997). During the task, participants lifted the 5 kg box (35.5 × 29 × 13.5 cm) between the shelves starting at the lower shelf. The speed of the task was controlled by a metronome with 2 s allocated to each movement between shelves followed by a rest of 2 s.

The task was standardized by using a universal weight of 5 kg for all participants, and providing identical instructions on how to complete the task, including instructions to prevent the movement of the feet and limit movement at the knee. These instructions were followed by a demonstration of the movement by a researcher, after which each participant was allowed to practice one timed movement with an unweighted box. Following familiarization with the task, the subjects completed a single cycle of lifting and lowering of the box.

This task was piloted extensively prior to the commencement of data collection to ensure that participants could complete the movement and LBP symptoms were monitored closely.

### Experimental Set-Up

#### Electromyography

**Figure 2:**
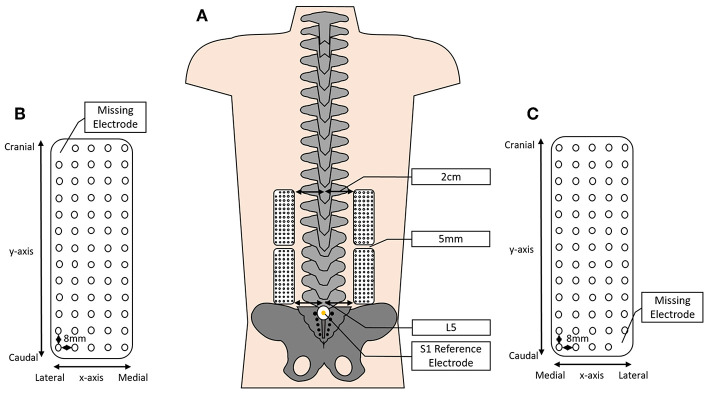
Depicting **(A)** the approximate positioning of the HDEMG grids over the lumbar and lumbothoracic erector spinae (ES), 2 cm lateral to the spinous processes and spaced 5 mm between grids; **(B,C)** a schematic of the HDEMG electrode grid depicting the inter-electrode distance, the positioning of the x- and y-axes and showing the location of the missing electrode (not to scale).

Surface HDEMG signals were recorded using four semi-disposable 13 × 5 2D electrode grids (OT Bioelettronica, Turin, Italy). Each electrode grid comprised of 64 electrodes spaced evenly with an 8 mm inter-electrode distance (Figure 2). The electrodes were adhered to the skin bilaterally, beginning at the level of the L5 spinous process and extending to approximately the level of T8–T10 depending on the height of the participant.

The electrode grids were prepared by first affixing a custom double-sided adhesive foam pad with holes for each electrode to the surface of the grid (SPES Medica, Genoa, Italy). The cavities created by the foam pad were filled with an electro-conductive paste. Before the electrodes were applied, the skin was prepared in order to reduce impedance and improve the quality of the bioelectrical signals recorded. First, the skin in the region that the electrodes would be placed was shaved and subsequently cleaned with an abrasive paste (SPES Medica, Genoa, Italy) and finally cleaned with water.

In line with previous studies, the lower grids were attached 5 cm lateral to the lumbar spinous processes, beginning at the level of L5 (Falla et al., 2014; Martinez-Valdes et al., 2019; Sanderson et al., 2019). The upper grids remained 5 cm lateral to the spinous processes; however, the inferior border was affixed to the skin 5 mm cranial to the superior border of the lower grid (Figure 2). Reference electrodes were placed on prepared skin overlying the S1 spinous process and the right medial malleolus.

EMG signals were sampled at 2,048 Hz and amplified by a factor of 150 (400 channel EMG Amplifier Quattrocento, OT Bioelettronica; −3 dB, bandwidth 10–500 Hz) before analog to digital conversion by a 16-bit converter. Recorded signals were saved on a computer hard drive and processed offline using custom MATLAB code (The Mathworks Inc., MA, USA). Each electrode grid recorded 64 monopolar signals, in this experiment two grids were used bilaterally equating to 128 monopolar signals per side. These signals were processed by filtering the signals using a 20–350 Hz 2nd order Butterworth bandpass filter (Gallina et al., 2011; Murillo et al., 2019), and the upper and lower grids combined at the intersection to create large left and right grids.

In order to best evaluate the distribution of muscle activity across this large region, monopolar signals were used for the following analysis. For each movement phase (S1-2 and S2-1), signals were divided into two equal epochs. The amplitude (RMS) was calculated for each signal and epoch, and these values were used to create topographical maps of the muscle activity. As described previously, the location of the centroid in the x- and y- axes was calculated from these topographical maps (Tucker et al., 2009; Falla et al., 2017; Sanderson et al., 2019). Additionally, values for the complexity (entropy) of the signals were calculated for each epoch, this measure indicates how homogenous the contraction was across the measured area, with a reduction in this value indicating a more heterogeneous contraction (Martinez-Valdes et al., 2019). The RMS and entropy were averaged across the large grids to obtain one value for each side.

#### Motion Analysis

**Figure 3:**
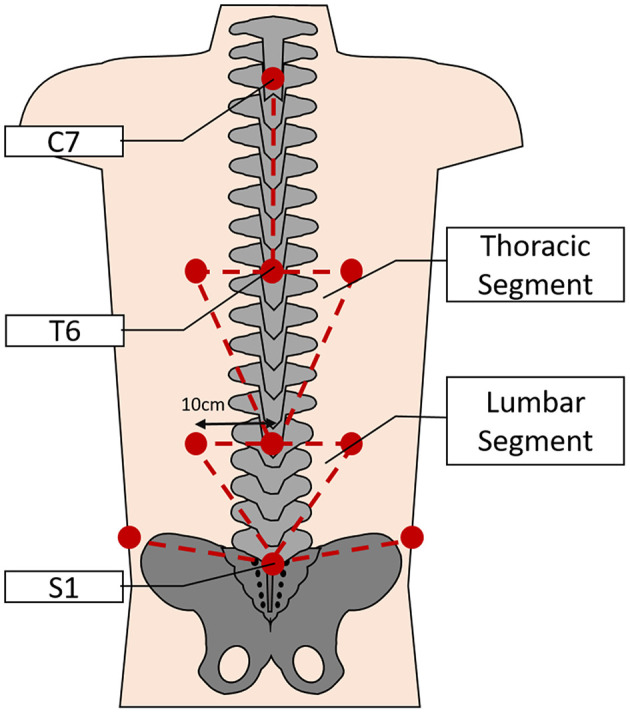
Depicting the approximate locations of the retroreflective markers on the back (lower limb markers not shown) and the subsequent segmental model used for analysis of spinal movements during the task (not to scale).

Three-dimensional kinematic data were recorded using an 8-camera stereo-photogrammetric array (Smart DX, BTS Bioengineering, Milan, Italy). Retroreflective markers were adhered on the participant's skin overlying anatomical landmarks which were identified by palpation. The landmarks chosen for this task included unilateral points on the spinous processes of C7, T6, T12, and S1; and bilaterally over the remaining landmarks; 10 cm lateral to the spinous processes of T6 and T12; the iliac crest on the mid-axillary line; the lateral femoral epicondyle; the lateral malleolus, the head of the fifth metatarsal and the calcaneal tuberosity. The pattern of marker placement on the back allows the spine to be split into functional areas as described previously by Muller et al. (2016). Thus, the markers overlying the spine and lateral areas were used to create the Lumbar Segment (LS) and Thoracic Segment (TS) (Figure 3). Finally, to correctly identify movement events throughout the task, additional markers were placed on the outside edge of each shelf and on the edge of the box which was to be lifted.

Throughout the lifting task, 3D kinematic data were recorded continuously and sampled at 150 Hz. A trigger signal from the HDEMG amplifier was recorded alongside the kinematic data in order to allow synchronization between the devices. All data were saved on a computer hard drive for offline processing.

Analysis of the 3D data was completed using the BTS SMART software suite and custom analysis protocols (SMART Tracker, SMART Analyser; BTS Bioengineering, Italy). Raw 3D data were tracked and labeled using custom kinematic models and any errors in the tracking were corrected to remove artifacts. Then, individual 3D data points were interpolated and then filtered using a 5 Hz Butterworth low-pass filter to ensure that each track was suitable for further analysis. Finally, the movements to each shelf were identified, and the 3D tracks were cut in order to separate data into movement from S1-2 and S2-1. For the spinal segmental analysis, a reference axis was created for each segment (TS, LS), which was applied to a virtual point in the center of the segment. The Euler angle was calculated between these axes in order to understand how the segments moved in relation to each other throughout the task.

### Statistical Analysis

Statistical analysis was completed using Statistica Version 13.3 (Tibco, USA), with an alpha level set at 0.5.

#### Sample Characteristics

Questionnaire responses were evaluated according to their respective guidelines (Waddell et al., 1993; Osman et al., 1997; Fairbank and Pynsent, 2000; Craig et al., 2003; Walsh et al., 2003; Henry and Crawford, 2005). Any differences between groups in demographic and questionnaire data were identified and tested for significance using student *t*-tests.

#### Motion Analysis

In this task, movement primarily occurred in the sagittal plane, thus for the motion analysis component of this study, the data considered involves only the movement around the horizontal axis in the sagittal plane. In order to understand differences in the movement strategy between groups, data were temporally normalized into 100 epochs representing 100% of the movement, and have been considered in terms of the shift from the first epoch. In order to achieve this, the angle between segments in the first epoch was considered as a zero-point, and was subtracted from each subsequent epoch, leaving just the deviation from epoch one. Any differences between groups for the movements were identified and tested for statistical significance using repeated measures ANOVA assessments with factors of group (LBP/CON) and time (100 epochs).

#### HDEMG

Analyses were conducted considering both absolute values (right and left) and, in line with several previous studies, comparing the most painful side for the LBP group to the right side of the control group (Falla et al., 2014; Sanderson et al., 2019). The choice of the right hand side for CON participants is reflective of previous investigations using this side, and of the mono-planar nature of the task.

Where no significant differences were identified between the first and second epochs of each movement, and thus to improve clarity in the results, the mean values across the pairs of epochs have been reported. Differences between groups were identified using factorial ANOVAs with factors of Group (LBP/Control), Side (Left/Right), Movement (S1-2/S2-1), and Epoch (Lifting/Lowering).

Topographical maps of the muscle activity were created from the absolute RMS values for each group. As the spatial distribution data were presented in the form of an image, only qualitative analysis was possible. The maps were classified by two independent raters in terms of the pattern of the distribution of activity. Each rater classified each map as either “diffuse activation,” “predominantly diffuse,” “cranial activation,” or “caudal activation.” Ratings were conducted independently and percentage agreement scores were calculated between raters (AS/EMV).

## Results

### Sample Demographics

Fourteen control (CON) and eleven LBP participants completed the lifting task. There were no significant differences in the demographic characteristics between groups, with no differences identified in the mean age, BMI or gender distribution between groups (all *P* > 0.05). The LBP reported a mean current pain level of 2.59/10 and ODI score of 16.1.

The questionnaire data revealed no significant differences in the level of physical activity between groups (measured by the IPAQ; *P* > 0.05) or the level of mental health (measured by the DASS-21 and mental component summary (MCS) portion of the SF-36; both *P* > 0.05). Significant differences were identified in measures related to back pain. The LBP group demonstrated higher levels of disability as measured by the ODI (*P* < 0.0001) and lower levels of physical health as measured by the physical component summary (PCS) portion of the SF-36 (*P* < 0.0001). Additionally, members of the LBP group showed higher levels of fear avoidance behaviors (*P* < 0.0001) and pain catastrophizing (*P* = 0.001) related to their LBP symptoms. Full demographic information from this sample is presented in Table 1.

**Table 1:** Sample demographics for the control and low back pain (LBP) groups showing standard deviations.

**Characteristic**		**Controls**	**LBP**	***P*-Value**
Sex		6 male, 8 female	5 male, 6 female	–
Mean age (Years)		27.36 ± 11.38	32.45 ± 16.27	–
BMI		23.13 ± 4.32	25.07 ± 2.58	–
ODI^*^		0.29 ± 1.07	16.1 ± 7.73	*P* < 0.001
PCS^*^		6.07 ± 7.50	19 ± 10.13	*P* = 0.001
IPAQ		72% (High)	82% (High)	–
DASS-21		9.29 ± 9.69	21.82 ± 29.59	–
FABQ^*^		3.38 ± 6.31	27.28 ± 11.31	*P* < 0.001
SF-36	PCS^*^	57.84 ± 3.95	47.75 ± 4.92	*P* < 0.001
	MCS	52.98 ± 3.16	46.33 ± 15.42	–
PNRS	Current pain	–	2.59 ± 1.64	–
	Average pain	–	3.36 ± 2.06	–

*Where significant differences exist between groups the name of the outcome has been marked with an asterisk and a P-Value is displayed. PCS, physical component summary; MCS, mental component summary*.

### Muscle Activity

**Figure 4:**
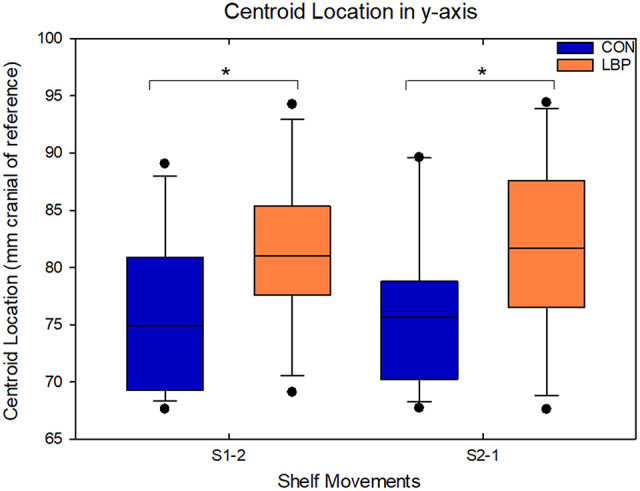
Demonstrating the location of the y–coordinate of the centroid in the craniocaudal axis for movements from both S1-2 and S2-1 within the absolute data; significant differences between groups are indicated with an asterisk.

In all analyses, there were significant systematic differences in the location of the centroid of muscle activity in the cranio-caudal direction (y-axis co-ordinate). Across both shelf movements, in both the analyses comparing the absolute data and the painful side analyses, the location of the y-coordinate of the centroid was systematically more cranial in the LBP group than in the control group. For the movement from S1-2, when comparing the absolute values, the average position for the y-coordinate of the centroid for the control group was 75.9 ± 8.4 mm cranial of the reference electrode, whereas for the LBP group, the centroid was 81.5 ± 7 mm cranial, a difference of 5.56 mm (*F* = 12.13, *P* = 0.0008). Similarly, for the movement from S2-1 the centroid was 76.1 ± 8.1 mm cranial for the control group whereas it was 82.6 ± 8.9 mm for the LBP group, a mean difference of 6.47 mm (*F* = 14.25, *P* = 0.0003; Figure 4). Similar results were identified in the comparison of the painful side and the right hand side for the control group. On average the y-coordinate of the centroid was 4.02 mm more cranial in the LBP group for the movement from S1-2 (*F* = 4.08, *P* = 0.049), and 5.56 mm more cranial for the movement from S2-1 (*F* = 5.71, *P* = 0.021).

**Figure 5:**
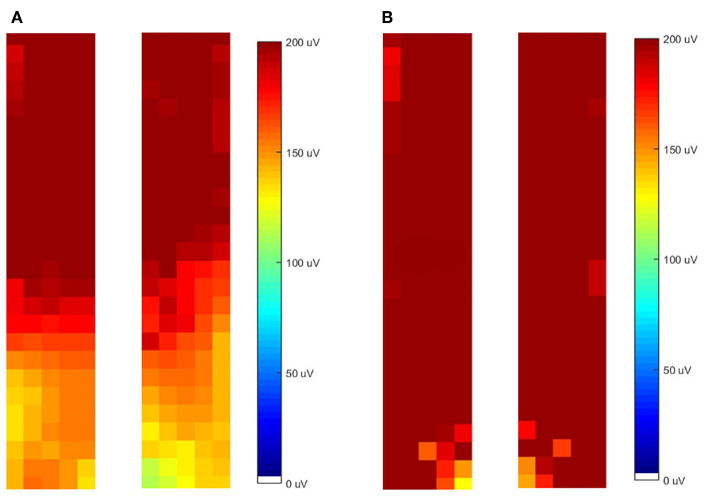
Representative examples of the topographical distribution of activity in the left and right grids for both low back pain (LBP) **(A)** and control (CON) **(B)** participants.

The difference in the spatial distribution of activity was reflected in the topographical maps of muscle activity across the lumbar and thoracolumbar ES. From observation of the maps, the LBP group displayed a more cranial distribution of activity, while the control group showed a more even distribution across the entire muscle (see Figure 5 for a representative example). When two individuals independently (R1/R2) classified the distribution of activity, for movement S1-2 the LBP group showed a more cranial distribution of activity in 7/11 (R1) or 9/11 (R2) cases whereas the CON group showed an even distribution across the region in 11/14 (R1) or 13/14 (R2) cases (84% reviewer agreement). Similarly, for the movement from S2-1 the LBP group demonstrated a more cranial distribution of activity in 6/11 (R1) or 8/11 (R2) cases and the CON group showed an even distribution in 12/14 (R1) or 11/14 (R2) cases (88% reviewer agreement).

**Figure 6:**
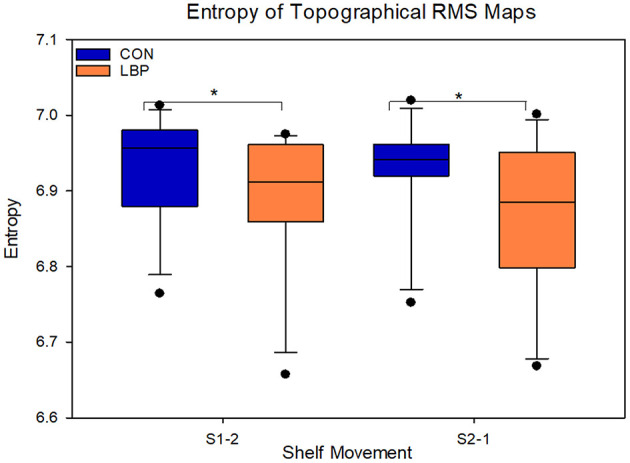
Depicting the entropy of the low back pain (LBP) and control (CON) groups. Significant differences are marked with an asterisk.

When comparing the absolute values for entropy to evaluate the homogeneity of muscle activity, the control group had systematically higher levels of entropy for both movement phases, indicating a more uniform distribution of activation across the EMG amplitude map. For the movement from S1-2, the control group had an entropy of 6.93 ± 0.09 and the LBP group had an entropy of 6.89 ± 0.11 (*F* = 4.7, *P* = 0.03). For the movement from S2-1 the CON group entropy remained at 6.93 ± 0.09, however the LBP entropy was lower again at 6.86 ± 0.13 (*F* = 8.3, *P* = 0.004; Figure 6).

**Figure 7:**
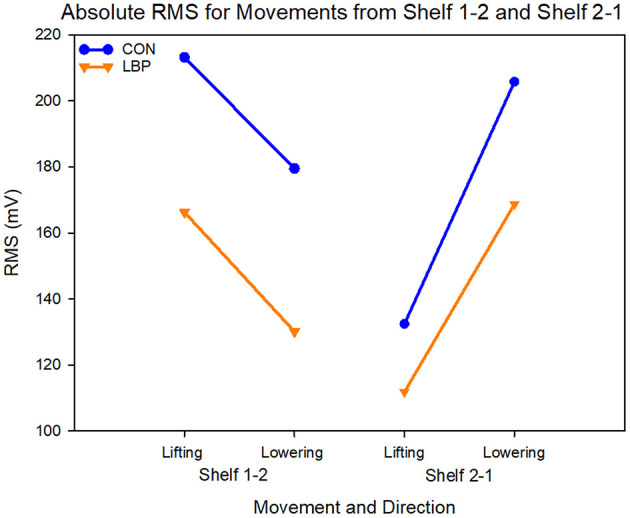
Graph showing the absolute values for RMS in mV for each shelf movement across both lifting and lowering epochs.

When comparing the mean results from each group, the LBP group also showed systematically lower RMS in both lifting directions. For S1-2, the average RMS for the control group was 48.14 mV greater at 196.33 ± 82.7 mV than the LBP group at 148.19 ± 57.1 mV (*F* = 11.22, *P* = 0.001). For the movement from S2-1 the mean control group RMS was 169.11 ± 75.6 mV and the LBP RMS was 140.27 ± 62.1 mV, a mean difference of 28.84 mV (*F* = 5.08, *P* = 0.026; Figure 7). In this task, both groups also showed a significant difference in the RMS between the lifting and lowering epoch for both movements (S1-2—*F* = 5.9, *P* = 0.02; S2-1—*F* = 26.0, *P* < 0.0001) however no group interactions were present.

No significant differences were identified between groups for the location of the centroid in the mediolateral x-axis in any condition. When comparing the most painful side of the LBP group to the right side of the control group, there were no differences in mean x-coordinate of the centroid throughout the task (*P* = 0.41); and no difference in the shift of the centroid in the x-axis throughout the lifting task (*P* = 0.43).

### Kinematics

**Figure 8:**
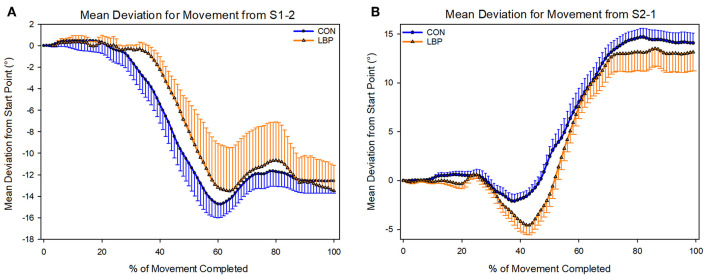
Depicting the mean deviations from the start position used by each group to complete each shelf movement (**A**—Shelf movement 1–2; **B**—Shelf movement 2–1).

There was no difference in the movement pattern used to complete the task. Participants in both groups used the same lumbar-thoracic segment movement pattern to complete both movements. No statistically significant differences were identified for the movement from S1-2 (*P* = 0.81) or from S2-1 (*P* = 0.24; Figure 8).

## Discussion

This study uniquely demonstrates that individuals with LBP utilize a more cranial and less diffuse distribution of ES activity during a singular mono-planar lifting task when compared to healthy controls, even while no difference in the kinematics of the thoraco-lumbar segments was observed. This study is the first to assess both lumbar and thoracolumbar ES contributions to a lifting task in both the painful and non-painful side in participants with LBP.

The participants recruited for this study were anthropomorphically comparable, with no significant differences in measures of BMI, age or biological sex between groups. Within the questionnaire results, there were also no differences between groups for the level of physical activity or for general mental health. However, understandably there were significant differences in measures designed to assess the impact of pain on day-to-day life. The LBP group reported higher levels of disability, pain catastrophizing and fear avoidance behaviors than the control group, consistent with previous research (Waddell et al., 1993; Fairbank and Pynsent, 2000; McWilliams et al., 2015). However, with the mean ODI score of 16/50 and average pain at the time of data collection of 2.6/10, it is suspected that this group has only mild to moderate levels of pain and were highly functional (Breivik et al., 2008).

Evidence from previous studies has shown that LBP participants demonstrated reduced range of motion in spinal flexion when compared to healthy controls (Falla et al., 2014; Shojaei et al., 2017). This is likely due to the controlled nature of the task, as the participants received a demonstration of the movement and then were allowed to practice with an unweighted box to ensure that they completed the task correctly. Furthermore, they were asked to complete the task to the beat of a metronome to facilitate standardization. While other studies have similar pre-task training, as this task was short, it is possible that participants did not have the time to alter their movement strategy as pain was provoked or myoelectric fatigue developed as may be the case in longer tasks, however the myoelectric manifestations of fatigue were not formally measured here. Therefore, if the task were to be sustained for several minutes then it is possible that differences may become apparent. Furthermore, while this task design has not been investigated in a LBP population, an investigation in a healthy cohort reported low variability in movement, indicating that a movement with few repetitions might be expected to have little variability between subjects (Peach et al., 1998).

Consistently within the results of this study, there was less diffuse and more cranially focused ES activity in the LBP group. The differences seen from the topographical EMG amplitude maps were supported by the statistically significant differences in the location of the y-coordinate of the centroid and entropy between groups. Specifically, those with LBP performed the lifting task with greater activity in cranial regions of the thoracolumbar ES relative to the lumbar ES, resulting in greater heterogeneity of activity across the entire region examined. These findings suggest that the LBP participants engaged lower thoracic portions of the ES, that is the *iliocostalis lumborum pars thoracis* or the *longissimus thoracis pars thoracis*, to complete a targeted lumbar lifting task, relative to asymptomatic people.

The cranial shift of activity seen in the LBP group relative to the control group, likely implies the utilization of less biomechanically favorable muscle fibers to complete the lifting task (Bogduk, 2005). The structure of the ES in the lumbar region is laminated. The deepest and most medial fibers of the ES connect L5 to the PSIS, with fibers to each subsequent lumbar vertebrae forming progressively more superficially and laterally (Bogduk, 1980, 2005; Christophy et al., 2012). Thus, activation focused in the more cranial portions of the muscle is representative of primarily using the fibers extending to the higher lumbar vertebrae rather than the whole muscle. In this task due to the weighted box, which was held anterior to the participant, the forces through the lumbar spine were multiplied when the participant leant forward to complete the task (Han et al., 1995). Previous studies which have investigated the effect of dynamic lifting on this region have reported increases in both the L5/S1 sheer forces and activity in the lumbar extensors (Arjmand and Shirazi-Adl, 2005). However, here we saw that activity was focused more cranially in LBP participants, potentially reducing the muscle fibers which were utilized to resist the increased sheer forces at the lower lumbar vertebrae.

The topographical redistribution of EMG activity within a muscle, during a fatiguing task, has also been observed in the trapezius muscle (Barbero et al., 2016). This phenomenon appeared to be correlated with the duration of the task and appears to be important to maintain the motor output (Farina et al., 2008). Notably, previous investigations including both dynamic and static tasks in presence of neck pain, consistently showed a redistribution of upper trapezius activity in a caudal direction (Falla et al., 2010, 2017). When considering the findings reported here, the variation in muscle activation occurs in a different manner but can be considered the output of the same adaptation strategy aimed to avoid pain or overload. Although, we cannot exclude that regional nociception can inhibit the muscle activity in some specific anatomical regions. In both cases the underlying physiological adaptations can play an important role in the clinical course of musculoskeletal pain.

## Strengths and Limitations

This study is the first to assess muscle activity comprehensively over the lumbar and thoracolumbar region of the ES bilaterally during a lifting task. This allowed for the measurement of the effect that occupational lifting can have on individuals who already experience LBP; granting a deeper insight into the distribution of activity across the entire muscle region, and allowing the identification of a differing activation pattern.

A relatively small sample was used for this study which might account for some of the variability within the data. Nevertheless, the sample size was sufficient to reveal significant differences between groups. Additionally, the symptomatic participants were highly functional and reported only mild levels of LBP and so it is unlikely that these results can be extrapolated to those with more severe pain or acute LBP. Therefore, it might be difficult to generalize these results to patients with more severe symptoms, however it is speculated that even greater differences might be identified in patient groups with more severe symptoms or longer pain duration (Mannion et al., 2000; Arendt-Nielsen and Graven-Nielsen, 2008).

Here we designed a task to assess the effect of lifting a light load by an individual who has LBP; however, most occupational lifting tasks would comprise of repeated lifting, as in a factory setting, or multidirectional lifting. Nevertheless, our focus was to comprehensively evaluate muscle behavior and spinal movement during a single lift, without the presence of myoelectric manifestations of fatigue or provoked pain which could confound interpretation of the results, although as fatigue was not measured here this is slightly speculative. One final potential limitation of this study is in the qualitative interpretation of the spatial distribution data. As both reviewers are investigators in the study, there is a potential source of bias based on prior expectations, however reviewers were blinded to the group of the participant, and reviewed independently with good levels of agreement.

## Conclusion

Participants with mild to moderate LBP utilize a different motor strategy to complete a lifting task which was characterized by a less diffuse, less homogenous, and more cranial focussed activation of the ES. This difference in muscle behavior was observed despite a consistent movement strategy between groups. This modified activation pattern identified within the LBP group could provide a basis for future treatments focussed upon retraining the lumbar musculature to ease LBP symptoms.

## Data Availability Statement

The datasets generated for this study will not be made publicly available as it includes confidential data.

## Ethics Statement

Ethical approval for this study was granted by the University of Birmingham Ethics Committee (ERN_16-1389B), the procedures followed the Declaration of Helsinki and are reported in line with the Strengthening the Reporting of Observational Studies in Epidemiology (STROBE) guidelines (Von Elm et al., 2008).

## Author Contributions

AS, EM-V, and DF contributed to the design of the study. AS and PK acquired the data. AS, CC, EM-V, DF, and MB performed the data analysis. Drafting of the manuscript was performed by AS and DF. All authors contributed to interpretation of the data, participated in revising the manuscript, and approving the final submission.

### Conflict of Interest

The authors declare that the research was conducted in the absence of any commercial or financial relationships that could be construed as a potential conflict of interest.
